# Xeno-free generation of human induced pluripotent stem cells from donor-matched fibroblasts isolated from dermal and oral tissues

**DOI:** 10.1186/s13287-023-03403-7

**Published:** 2023-08-09

**Authors:** Hassan R. W. Ali, Salwa Suliman, Tarig Al-Hadi Osman, Manuel Carrasco, Ove Bruland, Daniela-Elena Costea, Helge Ræder, Kamal Mustafa

**Affiliations:** 1 Department of Clinical Dentistry, Centre for Translational Oral Research (TOR), University of Bergen, 5009 Bergen, Norway; 2 Department of Clinical Medicine, University of Bergen, Bergen, Norway; 3 Centre for Cancer Biomarkers, University of Bergen, Bergen, Norway; 4 Department of Medical Genetics, Haukeland University Hospital, Bergen, Norway; 5 Gade Laboratory for Pathology, Haukeland University Hospital, Bergen, Norway; 6 Department of Clinical Science, University of Bergen, Bergen, Norway; 7 Department of Pediatrics, Haukeland University Hospital, Bergen, Norway

**Keywords:** Platelet lysate, Fetal bovine serum, Fibroblasts, Xenogenic, Pluripotency

## Abstract

**Background:**

Induced pluripotent stem cells (iPS) can be generated from various somatic cells and can subsequently be differentiated to multiple cell types of the body. This makes them highly promising for cellular therapy in regenerative medicine. However, to facilitate their clinical use and to ensure safety, iPS culturing protocols must be compliant with good manufacturing practice guidelines and devoid of xenogenic products. Therefore, we aimed to compare the efficiency of using humanized culture conditions, specifically human platelet lysate to fetal bovine serum, for iPS generation from different sources, and to evaluate their stemness.

**Methods:**

iPS were generated via a platelet lysate or fetal bovine serum-based culturing protocol from matched dermal, buccal and gingival human fibroblasts, isolated from healthy donors (*n* = 2) after informed consent, via episomal plasmid transfection. Pluripotency, genotype and phenotype of iPS, generated by both protocols, were then assessed by various methods.

**Results:**

More attempts were generally required to successfully reprogram xeno-free fibroblasts to iPS, as compared to xenogenic cultured fibroblasts. Furthermore, oral fibroblasts generally required more attempts for successful iPS generation as opposed to dermal fibroblasts. Morphologically, all iPS generated from fibroblasts formed tight colonies surrounded by a reflective “whitish” outer rim, typical for iPS. They also expressed pluripotency markers at both gene (*SOX2*, *OCT4*, *NANOG*) and protein level (SOX2, OCT4). Upon stimulation, all iPS showed ability to differentiate into the three primary germ layers via expression of lineage-specific markers for mesoderm (*MESP1*, *OSR1*, *HOPX*), endoderm (*GATA4*) and ectoderm (*PAX6*, *RAX*). Genome analysis revealed several amplifications and deletions within the chromosomes of each iPS type.

**Conclusions:**

The xeno-free protocol had a lower reprogramming efficiency compared to the standard xenogenic protocol. The oral fibroblasts generally proved to be more difficult to reprogram than dermal fibroblasts. Xeno-free dermal, buccal and gingival fibroblasts can successfully generate iPS with a comparable genotype/phenotype to their xenogenic counterparts.

**Supplementary Information:**

The online version contains supplementary material available at 10.1186/s13287-023-03403-7.

## Introduction

Mesenchymal stem cells (MSC) have long been instrumental in regenerative medicine due to their multipotency, ability to self-renew and high proliferative capacity [1–3]. Promising results utilizing MSC for bone regeneration in both preclinical and clinical settings have been reported recently [4–9]. However, due to a number of challenges surrounding MSC, the quest to exploring alternative sources, such as pluripotent cells, is crucial [10–12]. Embryonic stem cells (ESC) can be expanded indefinitely without undergoing replicative senescence or aging due to their high telomerase expression. The main property that sets ESC apart from other cells is their pluripotent nature, meaning they can give rise/differentiate to cells of the three primary germ layers (mesoderm, endoderm and ectoderm) [13]. With time, however, concerns were raised surrounding the use of ESC due to drawbacks associated with the isolation process, immunogenicity and risk of teratoma formation [13–15]. The discovery of induced pluripotent stem cells (iPS), artificially generated via genetic alteration/reprogramming of mature somatic cells, offered some advantages relative to ESC [16–18]. Similar to ESC, iPS express pluripotent markers, have unlimited proliferation potential and possess the ability to differentiate into the three primary germ layers [19, 20]. Furthermore, iPS can be generated autologously and/or from a selected genetic background [15, 19]. To gain pluripotency, the reprogramming process typically involves transfecting adult somatic cells with certain pluripotency markers. Yamanaka initially reprogrammed fibroblasts by using four transcription factors, OCT4, SOX2, KLF4 and c-MYC, also known as the OSKM factors, or the Yamanaka factors [16, 17]. Since then, other groups have successfully reprogrammed adult cells to iPS via the use of various different cocktails of transcription factors [18, 21]. A multitude of methods for delivering these factors have been developed, which include both integrating and non-integrating methods. Integrating methods, such as retro and lentiviral delivery methods, leave behind an undesirable footprint via integration of exogenous genetic material into the cell genome. Therefore, in order to make iPS more clinically applicable, non-integrating reprogramming methods have been developed with zero footprint [21, 22]. These non-integrating methods include, among others, the use of episomal plasmids for delivery. Studies have shown that the episomal vectors do not persist and are spontaneously lost after a period of time [21, 23]. Nevertheless, despite the absence of any signs of the original plasmids, other footprints are possible, including artificially introduced genetic alterations. Copy number variations (CNV), such as deletions, insertions and duplications, exist naturally as structural variants in the human genome [24]. Such sub-chromosomal aberrations, along with whole-chromosome aneuploidies, have been regularly reported in human pluripotent cells (both ESC and iPS), with iPS likely having a higher number of CNVs than ESC [25–27]. This could be due to the reprogramming process itself being associated with increased CNV levels in the early stages of the resultant iPS. The number and total size of these CNVs, however, decrease dramatically with continued propagation/passaging of iPS [28]. Fibroblasts, specifically dermal fibroblasts, were the first cell type to be utilized for iPS generation [17]. Since then, different somatic cells have been used to generate iPS including keratinocytes, blood cells, dental pulp stem cells and mesenchymal stem cells [29–33]. Fibroblasts remain the most commonly used cell type for iPS generation, as they are generally easy to obtain and handle, and are commercially available for research purposes. Various cell types from the oral cavity have been used for the generation of iPS, as cells can be easily collected during dental procedures, without the need for extra invasiveness. In addition, wounds in the oral cavity heal rapidly without scar formation and with minimal patient discomfort [34, 35]. Hence, transitioning to oral sources for iPS generation has the potential to be a valuable approach.

To comply with good manufacturing practice (GMP) guidelines, it is vital to create cell culture protocols that are safe and standardized. Currently, media supplemented with fetal bovine serum (FBS) is the most widely used method of cell expansion [36]. Despite its large scale and frequent use, FBS is associated with a multitude of ethical, scientific and safety issues. For instance, variations in serum composition result in batch-to-batch heterogeneity, causing morphological and phenotypical differences, ultimately affecting reproducibility of cell expansion protocols [37–39]. As a result, FBS production has come under great scrutiny, and there is an increasing demand and need for animal-free culture techniques, which would allow for a safer and more ethical practice. With that being said, xeno-free alternatives have been developed, and platelet lysate (PL) has emerged as a promising “GMP compliant” candidate to replace FBS. PL is typically prepared from platelet derivatives which contain and release high concentrations of growth factors. These growth factors aid in the expansion of cells in culture. Moreover, PL is generally pooled from multiple donors, reducing donor-based variations [40, 41]. These attributes make PL an attractive alternative to FBS as a supplement to expansion media.

A few studies have claimed to generate iPS from xeno-free conditions, yet their protocol either includes FBS media for the culture of the somatic cells prior to iPS generation, or they fail to mention the somatic cell culture method altogether [42–44]. As far as we know, only a handful of studies have previously generated iPS using an entirely xeno-free protocol [45–48]. However, no effort was made to compare iPS generated from xeno-free protocols to those generated from xenogenic ones. Due to the aforementioned challenges, it is important to implement protocols that comply with GMP guidelines, *i.e.*, xeno-free culturing protocols, while also utilizing easily accessible sources for iPS generation. Therefore, in this study we aimed to analyze and compare iPS generated from donor-matched fibroblasts from different sources and evaluate their stemness. Furthermore, the study was aimed at evaluating and comparing the efficiency of using humanized culture conditions, specifically PL to FBS, on the generation of potent iPS.

## Materials and methods

### Fibroblast source and culture

Dermal, buccal and gingival samples were acquired from two healthy voluntary donors (Donor 1 (D1); female aged 40–50, and donor 2 (D2); female aged 50–60), following informed consent. All three samples were collected from each donor; dermal samples were obtained from the anterior forearm, buccal samples from the inside of the cheek and gingival samples from the gingiva above the upper first molar. The specimens were transported in Dulbecco’s modified Eagle’s medium (DMEM) (Gibco, ThermoFisher Scientific, Massachusetts, USA) supplemented with 3% penicillin/streptomycin (GE, Healthcare) and immediately processed for fibroblast isolation. Briefly, fibroblasts were isolated via the enzymatic digestion protocol as previously described [49]. Following isolation, fibroblasts were cultured and expanded in DMEM supplemented with 5% PL (Blood Bank, Haukeland University Hospital, Bergen, Norway) or 10% FBS (Gibco, ThermoFisher Scientific, Massachusetts, USA, catalog number: 10270106), creating two different culture conditions for cell expansion. The cells cultured in FBS were supplemented with 1% penicillin/streptomycin (GE, Healthcare), while the cells in PL were supplemented with 1% penicillin/streptomycin and heparin at a concentration of 2 IU/ ml (LEO Pharma). The morphology of the cells was observed using Nikon’s Inverted Light Microscope ECLiPSE Ts2R-FL (NIKON INSTRUMENTS EUROPE B.V., Amsterdam, the Netherlands). The fibroblast cell lines were regularly checked for mycoplasma contamination, and all lines were free of contamination prior to transfection.

### Fibroblast reprogramming and iPS culture

Approximately 5—6 × 10^5^ cells (passage 7–10) were transfected (Nucleofector 2b Device, Lonza, Switzerland) with 1 µg of each of the three episomal reprogramming plasmids (*pCXLE-hOCT3/4-shp53*, *OCT4 & shRNA p53*; *pCXLE-kSK*, *SOX2 & KLF 4*; *pCXLE hUL*, *L-MYC & LIN28)* and plated onto a six-well plate containing either FBS or PL supplemented DMEM (Gibco, ThermoFisher Scientific). Upon reaching confluency, the cells were passaged onto a 10 cm dish precoated with Geltrex (Gibco, ThermoFisher Scientific). The following day, culture media was changed to StemFlex media (StemFlex Medium, Gibco, ThermoFisher Scientific). Media was then changed every 1–2 days until stable colonies began to appear. Colonies were deemed stable upon formation of compact, round colonies with distinct borders [50]. Three colonies were then transferred to a Geltrex (Gibco, ThermoFisher Scientific) coated well in a 24-well plate, one colony per well. Each iPS colony was cultured individually in StemFlex media (StemFlex Medium, Gibco) and considered to be a biological replica. Gentle Cell Dissociation Reagent (Stem Cell Technologies, Vancouver, Canada) was used for cell detachment for passaging. Characterization and analyses of iPS were performed after passage 15. Cell morphology was observed using Nikon’s Inverted Light Microscope ECLiPSE Ts2R-FL (NIKON INSTRUMENTS EUROPE B.V., Amsterdam, the Netherlands). The iPS were regularly checked for mycoplasma contamination, and all lines were free of contamination prior to analysis.

### iPS characterization

#### Trilineage differentiation

The iPS were subjected to directed differentiation using STEMdiff Trilineage Differentiation Kit (Stem Cell Technologies, Vancouver, Canada). Briefly, iPS were detached from well plates using Gentle Cell Dissociation Reagent (Stem Cell Technologies) and centrifuged at 300 g for 5 min. The pellet was then resuspended in 1 ml of Single Cell Plating Medium according to the manufacturer’s instructions. The cells (8 × 10^5^ cells/well for endoderm and ectoderm differentiation and 2 × 10^5^/well for mesoderm differentiation) were transferred to a Geltrex coated 12-well plate. The media was then changed daily for 5 days (mesoderm and endoderm) and 7 days (ectoderm).

#### iPS Gene expression analysis (RT-PCR)

The pluripotency of the iPS along with their ability to differentiate into the three primary germ layers was assessed via quantitative real-time PCR (qPCR). Total RNA was isolated using a tissue RNA isolation kit (Maxwell, Promega, WI, USA), and a NanoDrop spectrophotometer (ThermoScientific, Delaware, USA) was used to check the quantity and purity of the isolated RNA. Total RNA (300 ng) was reverse transcribed, according to the manufacturer’s instructions, using a high-capacity complementary DNA reverse transcription kit (Applied Biosystems, CA, USA). qPCR was performed on a StepOne Plus system, using TaqMan gene expression assays (Applied Biosystems), to quantify the gene expression of pluripotency markers (*SOX2, OCT4, NANOG*) and trilineage markers for mesoderm (*MESP1, OSR1, HOPX*), endoderm (*GATA4*) and ectoderm (*PAX6, RAX*) lineages. Data were analyzed using the *∆∆Ct* method. Gene expression was normalized to that of the housekeeping gene, *GAPDH*. Expression of pluripotency markers is presented as fold changes relative to the control, dermal fibroblasts (DF) in FBS. Expression of trilineage markers is presented as fold changes relative to uninduced DF-iPS in FBS. An overview of the primers used for the gene expression analysis is presented in Additional file 1.

#### Flow cytometry

The iPS phenotype was analyzed via flow cytometry for specific markers, namely SOX2 and OCT4 (BD Biosciences, San Jose, CA, USA), according to the manufacturer’s instructions. The cells (~ 5 × 10^5^) were fixed in 10% buffered formalin, for 10 min, and permeabilized via 0.1% Triton X, 15 min in the dark. The pellet was then blocked in 0.5% bovine serum albumin, BSA (Sigma-Aldrich, St. Louis, MO, USA), in phosphate-buffered saline (PBS), for 10 min at room temperature. Conjugated monoclonal antibodies were then added to the pellet, and the cells were incubated in the dark for 30 min at 4 °C. The cells were then washed with PBS before being resuspended in PBS. Stained samples were analyzed and compared to the corresponding unstained samples (negative control). The final quantification was performed with a BD Accuri flow cytometer (BD Biosciences), and the data were analyzed using FlowJo (FlowJo, LLC, Ashland, OR, USA).

#### Chromosome microarray analysis

Whole-genome high-resolution chromosome microarray analysis was performed using the Applied Biosystems CytoScan HD Array Kit and Reagent Kit Bundle (Applied Biosystems Catalog number: 901835) according to the manufacturer's protocol. Briefly, 250 ng of genomic DNA was digested with the restriction enzyme NspI and then ligated to an adapter, followed by PCR amplification using a single pair of adapter primers. The PCR products were purified using magnetic beads (Agencourt AMPure, Beckman Coulter, Beverly, MA). Purified PCR products were then fragmented using DNase I, and the fragmented PCR products end-labeled with biotin and then hybridized to the array using the Affymetrix GeneChip Hybridization Oven 645 (Affymetrix Inc., USA). Arrays were washed and stained using a GeneChip Fluidics Station 250 and scanned using an Affymetrix GeneChip Scanner 3000 7G (Affymetrix Inc.). Scanned data files were generated using Affymetrix GeneChip Command Console Software, version 4.1, and analyzed with Affymetrix Chromosome Analysis Suite version 4.2.1 (ChAS) (Affymetrix Inc.) and BENCH Lab CNV—version 5.1.12 (Agilent Technologies, USA). Filtration was performed against a list of common abbreviations acquired from Affymetrix. Duplications were filtered if at least 90% overlap, containing at least 80 markers and listed at least 25 times in the list of common abbreviations. Deletions were filtered if 90% overlap, containing at least 30 markers and listed at least 25 times in the list of common abbreviations. LSCH regions less than 5 Mbp or supported by less than 500 markers were filtered.

### Ethical approval

Approval was granted by the Ethical Committee for Medical and Health Related Research in West Norway (REK 80005). Tissue samples were collected from two healthy voluntary donors after obtaining informed consent.

### Data presentation and statistical analysis

Statistical analysis was performed via IBM SPSS Statistics (SPSS Inc.). Data are presented as mean values (± standard deviation). Statistical significance was determined via an independent samples T-test when comparing two groups and one-way analysis of variance (ANOVA) when comparing more than two groups. A *p*-value of < 0.05 was considered statistically significant.

## Results

### Isolation of fibroblasts in xeno-free conditions

**Fig. 1:**
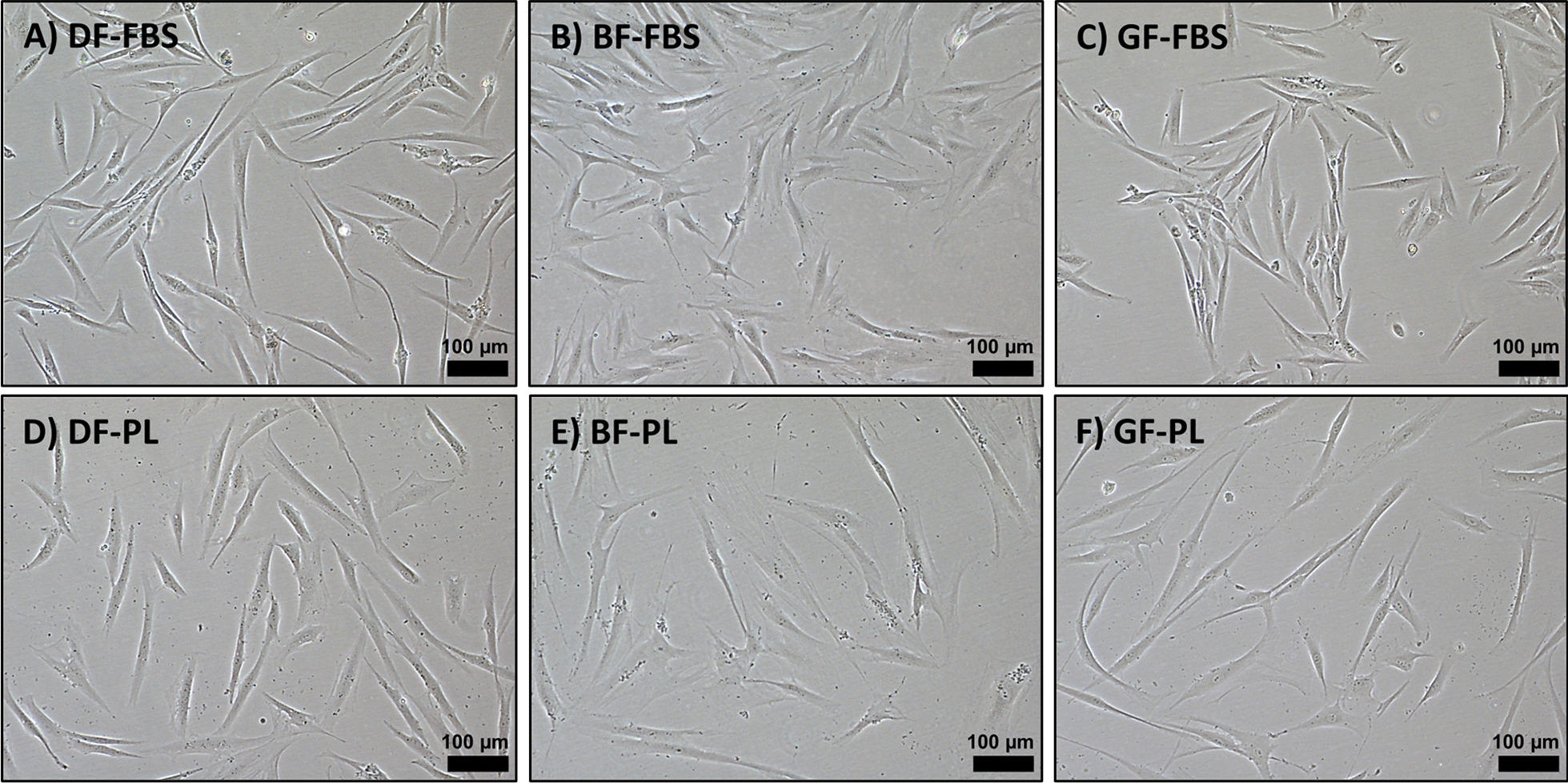
Representative light microscopy images demonstrating the morphology of the fibroblasts from different sources expanded in FBS (A–C) and PL (D–F). Scale bar: 100 µm (DF: dermal fibroblasts, BF: buccal fibroblasts, GF: gingival fibroblasts, FBS: fetal bovine serum, PL: platelet lysate)

The isolated matched dermal, buccal (BF) and gingival (GF) fibroblasts displayed a spindle-shaped morphology (Fig. 1).

### Generation of xeno-free iPS

**Fig. 2:**
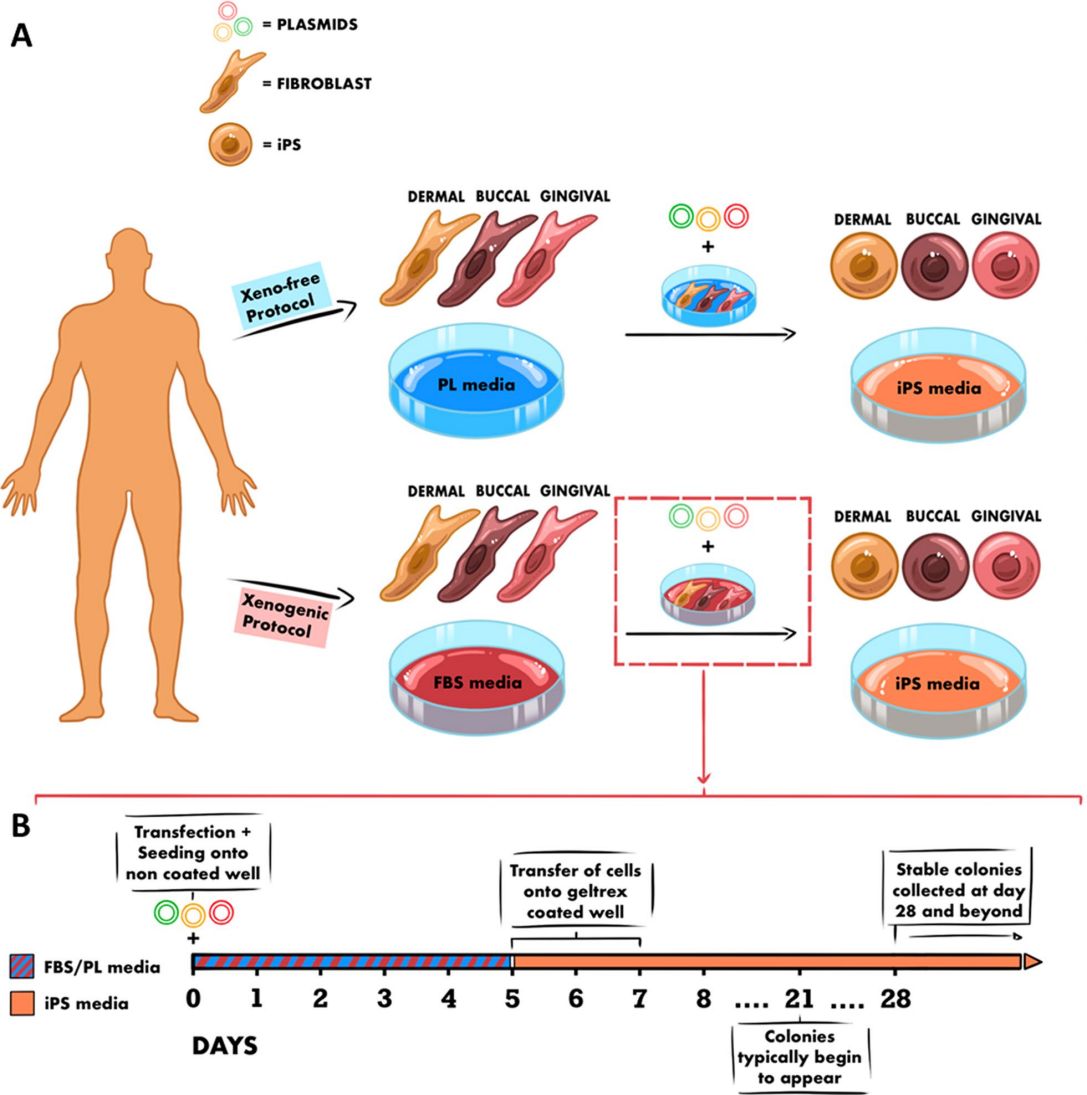
**A** Diagram illustrating the xeno/xeno-free generation of iPS, via episomal plasmid transfection, from different sources of matched fibroblasts. **B** Detailed illustration of the transfection/reprogramming procedure. This figure was created using Procreate 5.2 on iOS software (iPS: induced pluripotent stem cells, FBS: fetal bovine serum, PL: platelet lysate)

Matched DF, BF and GF from 2 donors were reprogrammed into iPS. The protocols for generating iPS from the different fibroblasts are illustrated in Fig. 2. The maximum number of reprogramming attempts was set at nine, after which the success rate (based on the number of attempts required to successfully develop iPS colonies) was determined. All the iPS reprogrammed successfully with the exception of one sample of gingival fibroblasts isolated in PL (GF-PL, D2). Fibroblasts isolated and grown in FBS generally showed a higher reprogramming success rate than those in PL. In most cases, DF showed the highest reprogramming success rate, while BF and GF showed varying success rates with no clear pattern (Table 1). Due to the BF-PL from D2 not yielding any iPS, the characterization of the iPS in this study was limited to the those obtained from D1.

**Table 1:** Data on the reprogramming of fibroblasts expanded in FBS/PL supplemented media

Fibroblasts	Success rate (number of reprogramming procedures attempted)		Successful reprogramming		First day of iPS colony collection	
DONOR 1	FBS	PL	FBS	PL	FBS	PL
DERMAL	100% (1)	33% (3)	Yes	Yes	32	33
BUCCAL	100% (1)	50% (2)	Yes	Yes	34	35
GINGIVAL	100% (1)	11% (9)	Yes	Yes	32	39

Fibroblasts	Success rate (number of reprogramming procedures attempted)		Successful reprogramming		First day of iPS colony collection	
DONOR 2	FBS	PL	FBS	PL	FBS	PL
DERMAL	50% (2)	33% (3)	Yes	Yes	28	31
BUCCAL	11% (9)	0% (9)	Yes	N/A	77	N/A
GINGIVAL	20% (5)	25% (4)	Yes	Yes	31	62

### Stemness of the generated iPS

**Fig. 3:**
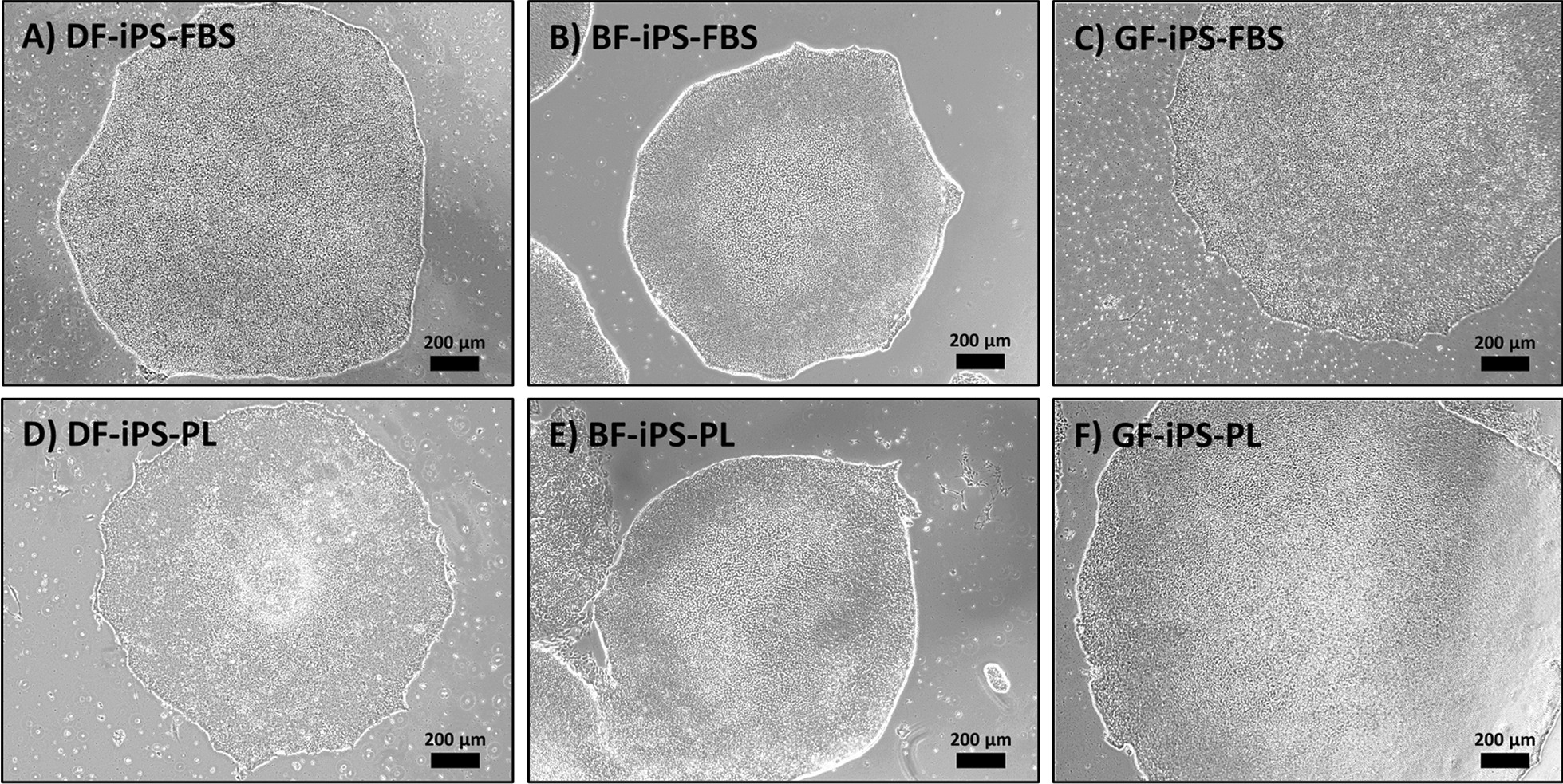
Representative light microscopy images demonstrating the morphology of the iPS from different sources expanded in FBS (A–C) and PL (D–F). Scale bar: 200 µm (DF: dermal fibroblasts, BF: buccal fibroblasts, GF: gingival fibroblasts, iPS: induced pluripotent stem cells, FBS: fetal bovine serum, PL: platelet lysate)

All the reprogrammed fibroblasts developed a stable colony morphology resembling ESC [50]. Morphologically, the cells grew in colonies, surrounded by a reflective “whitish” border, that increased in size as the cells proliferated, ultimately fusing with other colonies in the same well (Fig. 3).

#### Generated iPS express pluripotent genes

**Fig. 4:**
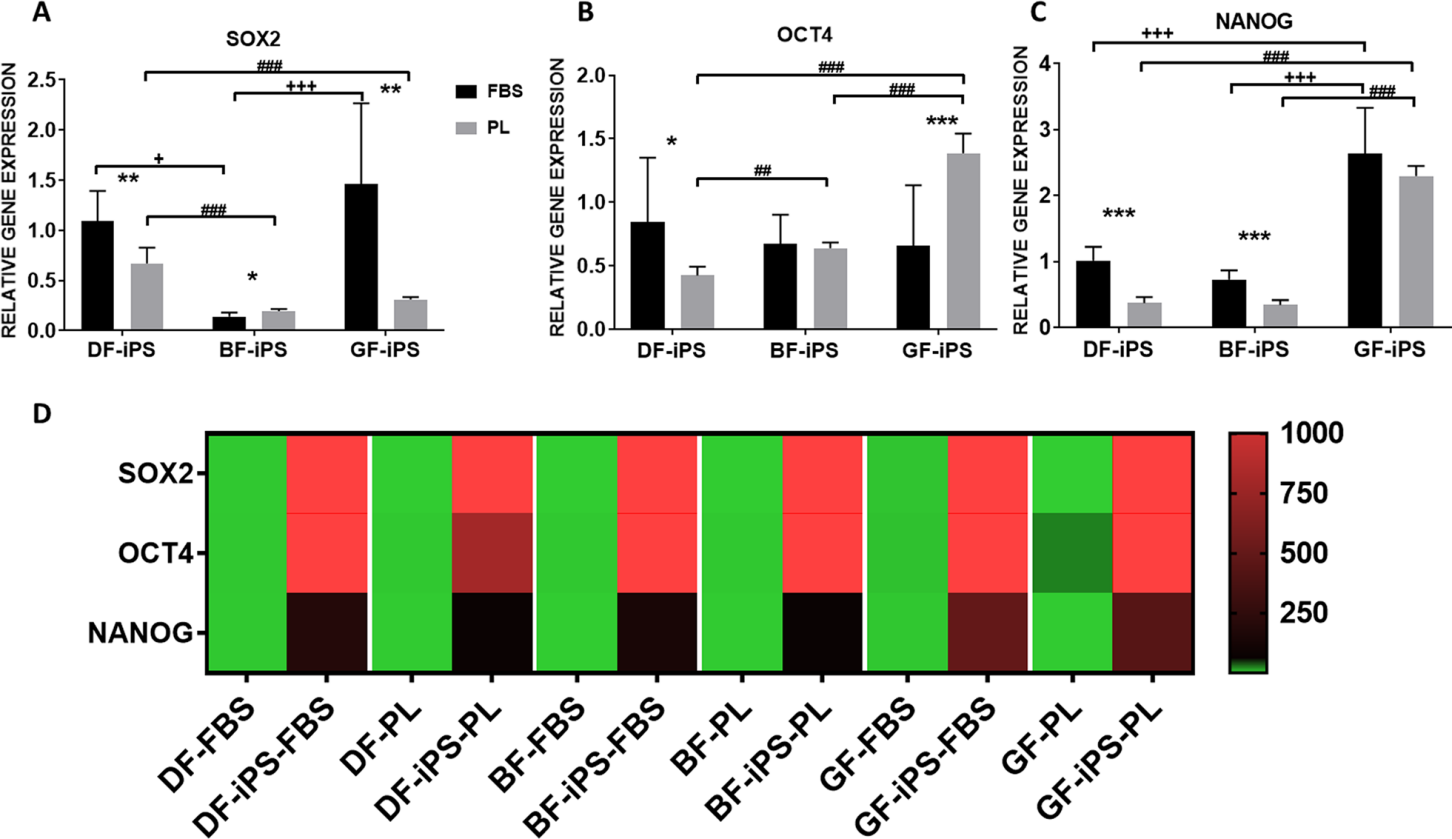
Relative gene expression of **A** *SOX2* **B** *OCT4* and **C)** *NANOG* by iPS in FBS/PL. Expression is presented relative to the DF-iPS-FBS group ± SD. **D** Heatmap analysis of the gene expression of *SOX2*, *OCT4* and *NANOG* by the fibroblasts in FBS/PL and their resultant iPS. Expression presented relative to the DF-FBS group. Independent samples t-test and one-way ANOVA were used to determine statistical significance (*p* < 0.05). (*) represents significance between iPS, from the same source, grown in FBS to those grown in PL. (+) represents significance between the iPS-FBS. (#) represents significance between the iPS-PL (DF: dermal fibroblasts, BF: buccal fibroblasts, GF: gingival fibroblasts, iPS: induced pluripotent stem cells, FBS: fetal bovine serum, PL: platelet lysate)

Gene expression analysis showed that the iPS expressed significantly higher levels (*p* < 0.001) of the pluripotency markers, *SOX2*, *OCT4* and *NANOG*, than their respective controls (undifferentiated fibroblasts) which showed little to no expression of these markers (Fig. 4). The iPS-FBS generally displayed higher levels of these pluripotency markers than their matched iPS-PL (Fig. 4).

Within the iPS-FBS, the GF-iPS revealed higher expression of *SOX2* and *NANOG* than both BF-iPS (significantly higher) and DF-iPS (significantly higher in the case of *NANOG*). Expression of *OCT4* by the iPS-FBS was comparable. The DF-iPS in turn revealed higher expression of *SOX2* (significantly higher) and *NANOG* than the BF-iPS, and slightly higher expression of *OCT4* than both the oral iPS. Within the iPS-PL, the GF-iPS showed a significantly higher expression of *OCT4* and *NANOG* than both the DF-iPS and BF-iPS, which in turn showed comparable expression. *OCT4* expression was also significantly higher in the BF-iPS when compared to the DF-iPS. *SOX2* expression was significantly higher in the DF-iPS than both the BF-iPS and GF-iPS (comparable).

#### Proteomics of pluripotency markers

**Fig. 5:**
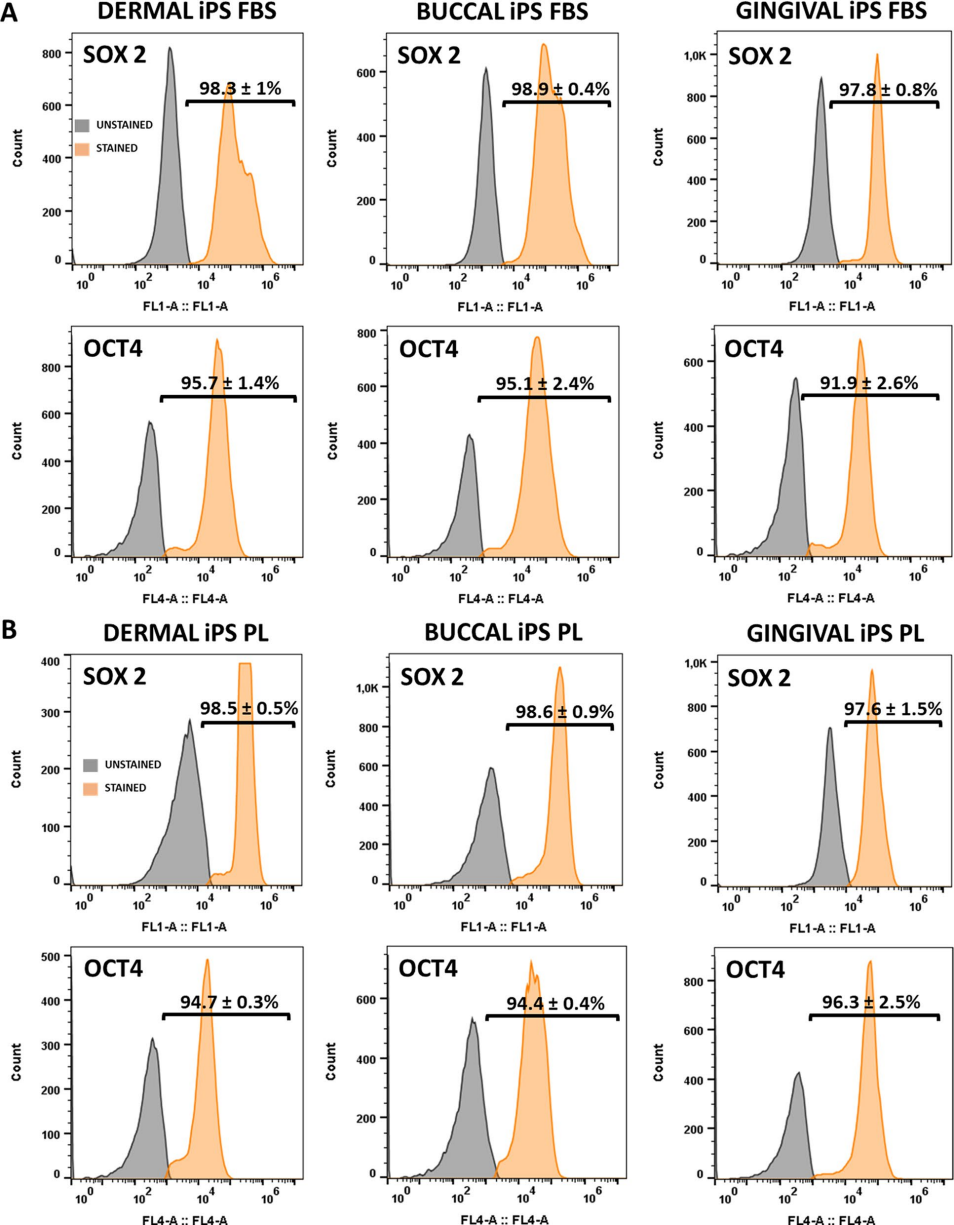
Flow cytometric analysis of **A** iPS-FBS and **B** iPS-PL showing detection of intracellular pluripotent markers SOX2 and OCT4 (percentage averages ± standard deviation) (iPS: induced pluripotent stem cells, FBS: fetal bovine serum, PL: platelet lysate)

Flow cytometric analysis generally revealed SOX2 and OCT4 positive iPS-FBS and iPS-PL. Analysis of the iPS-FBS revealed > 97% of cells positive for SOX2 and > 91% positive for OCT4. Analysis of the iPS-PL revealed > 97% of cells positive for SOX2 and > 94% positive for OCT4 (Fig. 5).

#### Trilineage differentiation ability of iPS

**Fig. 6:**
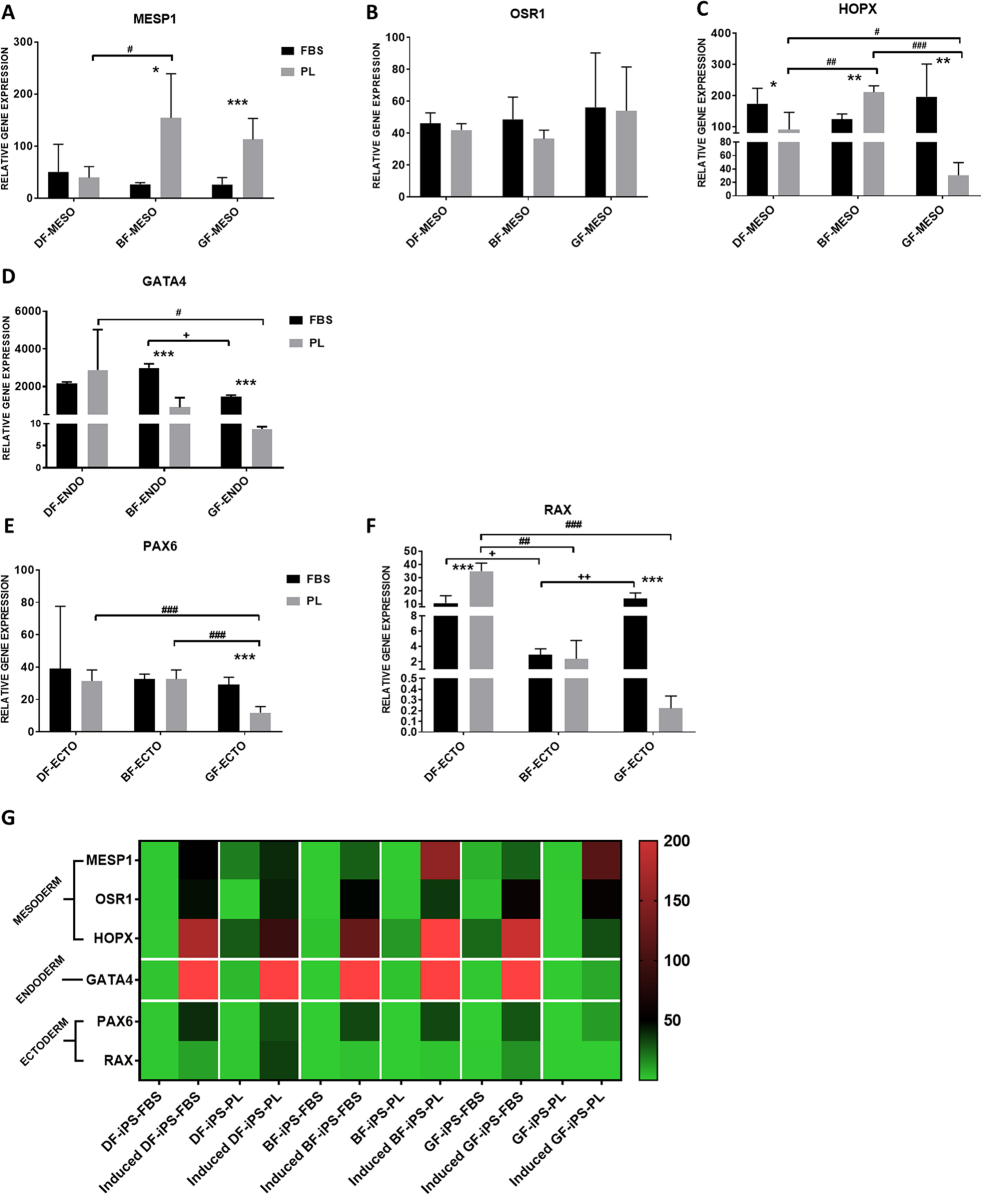
Relative gene expression of the **A–C** mesoderm, **D** endoderm and **E, F** ectoderm markers by the iPS following directed differentiation toward the three lineages. **G** Heatmap analysis of the gene expression of trilineage markers by the iPS before and after directed (induced) differentiation. Expression is presented relative to the uninduced DF-iPS-FBS group ± SD (uninduced iPS not shown on graphs). Independent samples t-test and one-way ANOVA were used to determine statistical significance (*p* < 0.05). (*) represents significance between the induced iPS, from the same source, grown in FBS to those grown in PL. ( +) represents significance between the induced iPS-FBS*.* (#) represents significance between the induced iPS-PL (DF: dermal fibroblasts, BF: buccal fibroblasts, GF: gingival fibroblasts, iPS: induced pluripotent stem cells, FBS: fetal bovine serum, PL: platelet lysate, ENDO: endoderm, MESO: mesoderm, ECTO: ectoderm)

Upon stimulation, all iPS showed ability to differentiate into the three germ layers, via expression of lineage-specific markers for mesoderm (*MESP1*, *OSR1*, *HOPX*) (Fig. 6A–C), endoderm (*GATA4*) (Fig. 6D) and ectoderm (*PAX6*, *RAX*) markers (Fig. 6E, F). Induced iPS showed significantly (*p* < 0.05) higher expression of the trilineage markers than their respective controls (undifferentiated iPS).

#### Mesoderm

Induced iPS-PL expressed *MESP1* at a significantly higher rate than their matched FBS, except in the case of DF-iPS where the expression was comparable. Expression of *OSR1* was comparable between the induced iPS-FBS and iPS-PL. Induced iPS-FBS revealed significantly higher expression of *HOPX* than their respective matched iPS-PL, except in the case of the BF-iPS where the iPS-PL displayed significantly higher expression than the iPS-FBS.

Within the induced iPS-FBS, expression of mesoderm markers was generally comparable throughout. Within the induced iPS-PL, *MESP1* expression was higher in the oral iPS than the DF-iPS, while *HOPX* expression by the DF-iPS and BF-iPS was significantly higher than the GF-iPS. The expression of *OSR1* was comparable in the iPS-PL.

#### Endoderm

Induced iPS-FBS expressed significantly higher levels of *GATA4* than their matched iPS-PL, except in the case of the DF-iPS where expression was slightly higher in the iPS-PL. Within the induced iPS-FBS, the BF-iPS expressed higher level of *GATA4* than both the DF-iPS and the GF-iPS (significantly higher), with the DF-iPS expressing higher levels than the GF-iPS. In the PL group, the DF-iPS showed higher *GATA4* expression than both the BF-iPS and the GF-iPS (significantly higher), with the BF-iPS expressing higher levels than the GF-iPS.

#### Ectoderm

Induced iPS-FBS expressed higher levels of *PAX6* than their matched iPS-PL (significantly higher in the case of the GF-iPS), except in the case of BF-iPS where expression was comparable. *RAX* expression was higher in the iPS-FBS than their matched iPS-PL (significantly higher in the case of GF-iPS), except in the case of the DF-iPS where the iPS-PL displayed significantly higher expression levels.

Within the iPS-FBS, *PAX6* expression was comparable. The DF-iPS and GF-iPS showed comparable expression levels and significantly higher levels than the BF-iPS. Within the induced iPS-PL, *PAX6* expression by the GF-iPS was downregulated compared to the DF-iPS and BF-iPS, which showed comparable expression. *RAX* expression was highest in the DF-iPS, followed by the BF-iPS.

### Genetic stability of iPS

**Fig. 7:**
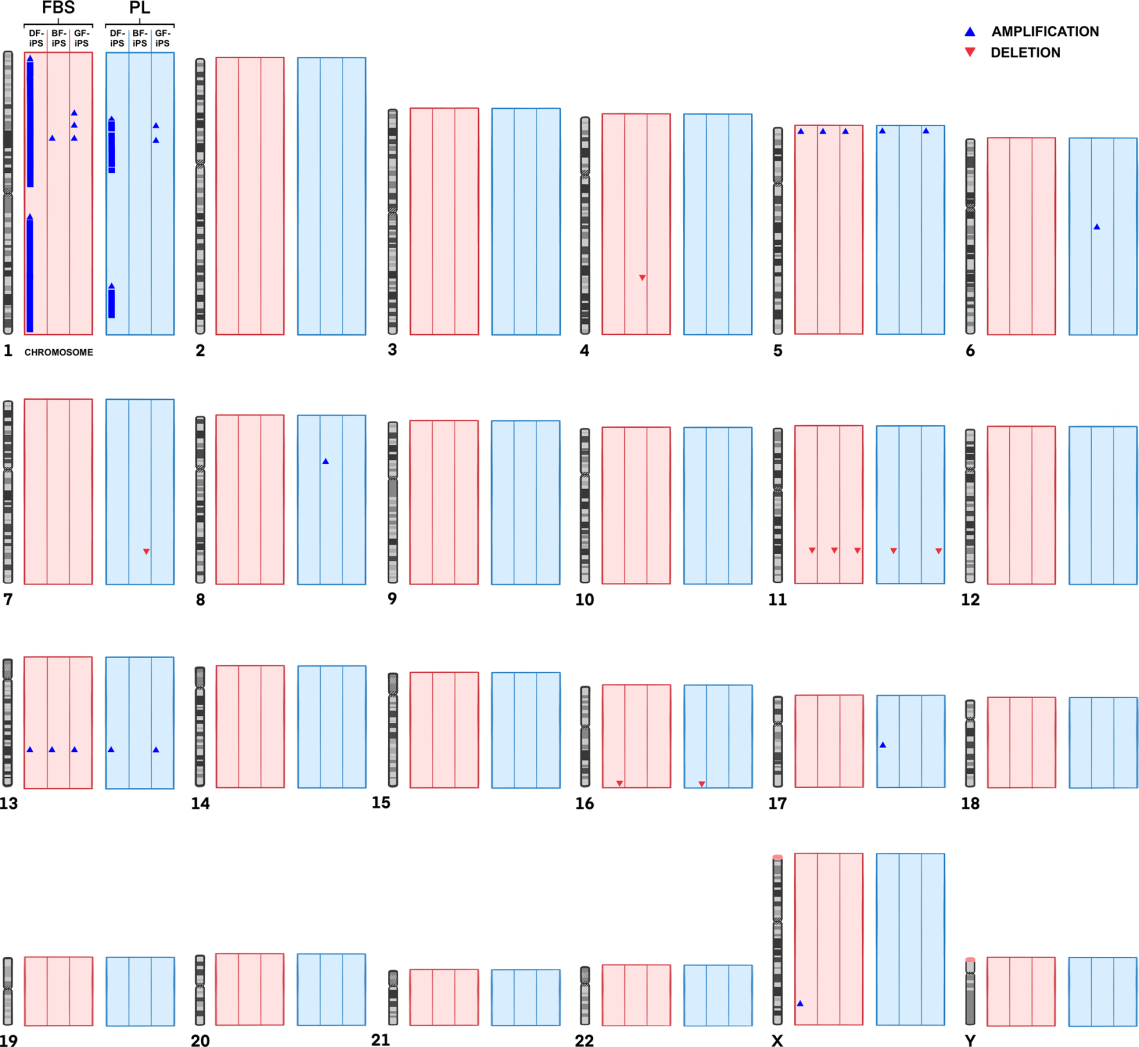
Representative* figure displaying the chromosomal CNVs of the different iPS-FBS and iPS-PL. The arrows/bars represent the gain (blue) or loss (red) of a chromosomal region. *Intended only as a representative figure and not for displaying exact locations of each CNV (DF: dermal fibroblasts, BF: buccal fibroblasts, GF: gingival fibroblasts, iPS: induced pluripotent stem cells, FBS: fetal bovine serum, PL: platelet lysate)

Chromosomal analysis revealed multiple amplifications and deletions within the genome of the iPS (Fig. 7). The iPS-FBS showed amplifications in chromosome 1, 5, 13 and X, and deletions in chromosomes 4, 11 and 16. The iPS-PL showed amplifications in chromosomes 1, 5, 6, 8, 13 and 17, and deletions in chromosomes 7, 11 and 16. A detailed genomic analysis of the iPS, including size and locations of the CNVs, can be found in Additional file 2.

## Discussion

To comply with GMP guidelines, cells must be cultured in xeno-free conditions prior to their use for iPS generation. To our knowledge, only a handful of studies have generated iPS from entirely xeno-free conditions [45–48]. Additionally, in most cases, no effort was made to compare iPS generated via xeno-free protocols to those generated in xenogenic ones. This paper is strengthened by the standardized conditions that the cells were subjected to throughout the entirety of the project. Donor-matched fibroblasts were cultured in xeno-free PL supplemented media and separately in xenogenic FBS supplemented media, simultaneously, from the time of isolation up until 1 week post-transfection. In addition, the fibroblasts were all transfected with the same cocktail of transcription factors, via the same method of delivery and in the same laboratory. These standardized conditions allow for efficient comparisons between xenogenic and xeno-free fibroblasts in the generation of iPS.

In this study, the reprogramming of donor-matched DF, BF and GF from two donors was attempted. The attempts, however, were not always successful, as presented in Table 1. Chow et al. also reported similar difficulties in obtaining iPS colonies from adult canine DF [51]. Such difficulties generally represent one of the major drawbacks associated with iPS production, as the efficiency of reprogramming somatic cells is deemed to be quite low, with efficiency levels as low as 0.0006% [22]. Several theories have been postulated in attempts to explain why only a small portion of transduced cells gain pluripotency. The general consensus is that cell reprogramming comprises two main phases: a primary stochastic phase and a secondary more deterministic phase [20]. Completion of both phases appears to be a rare event for most cells, hence the low reprogramming efficiency levels. With that being said, it is reasonable to expect that in some cases the reprogramming cycle would fail altogether. From the data presented here, it seems that a transition to a PL culturing protocol was less supportive of fibroblast reprogramming to iPS. A previous report from Sung et al. corroborates our results, where in their study, PL was also found to be less efficient at inducing cell reprogramming of human amniotic fluid stem cells. Reprogramming efficiency was also found to be significantly higher in the cells cultured in FBS supplemented media [46]. The literature has shown that PL culturing protocols do indeed affect the behavior of other cell types as well. For instance, PL has been reported to increase fibroblast proliferation rates compared to FBS [52]. In MSC, both disruption and maintenance of the undifferentiated cell state have been reported to be induced by PL [7, 53–55]. These conflicting reports could be attributed to the heterogeneity that exists between PL batches, due to the pooling of the blood derivatives from multiple donors [56]. Such PL-associated changes may likely have an effect on the behavior of fibroblasts and in turn affect their ability to differentiate to iPS.

When comparing cells from multiple individuals, despite the cell type being the same, donor variability must be accounted for. Cells obtained from different individuals tend to behave differently, both morphologically and functionally [57]. Similarly, inter-donor disparities, among other factors, may lead to variations among iPS [50, 58, 59]. For instance, cells obtained from the elderly are associated with an increased risk of iPS abnormalities and a decrease in reprogramming efficiency [60–62]. The results from this study clearly show different cellular responses, between the two donors, to the transfection procedure. For example, the BF from D1 (FBS/PL—100% success rate) were highly susceptible to reprogramming compared to the BF from D2 (FBS—33% success rate, PL—0% success rate). Besides donor variation, such differences may also be attributed to CNVs that might be acquired during the fibroblast reprogramming process [63]. These findings advocate for further investigations on the effect of donor variability on cellular reprogramming/iPS generation.

Certain elements must be considered when attempting to select the optimal cell source for reprogramming purposes, including invasiveness of the surgical procedure, ease of isolation and maintenance, and susceptibility to the reprogramming process [64]. Different somatic cell types have displayed varying results in terms of reprogramming susceptibility. Studies have revealed, for example, that keratinocytes are more easily reprogrammed than fibroblasts and that dental pulp stem cells yield more iPS colonies than bone marrow MSC [30, 33]. This disparity in susceptibility makes selection of the ideal cell source for reprogramming quite difficult. In this study, we found that DF are generally easier to reprogram than BF and GF. Yan et al. also reported similar difficulties when attempting to reprogram GF, with various transfection protocols yielding no iPS colonies [33]. It is not clear in their study, however, how many attempts were made to generate iPS from the GF. As it is with the GF in this study, it might be that continued attempts would have eventually led to the development of pluripotent colonies. The exact reasons as to why oral fibroblasts reprogram less efficiently than DF are unclear. However, inherent phenotypical differences between the fibroblasts are likely to play a role in reprogramming efficiency [65, 66]. Further investigation is required to determine the correlation and effect such innate characteristics have on the reprogramming process.

Due the BF-PL from D2 not yielding any iPS colonies, the focus was shifted to the results obtained from the analysis of D1. This allows for a more efficient comparison of the effect that different sources and culture conditions have on iPS generation. According to the literature, there are different levels of pluripotency, and cells should fulfill certain criteria at each level before being deemed as pluripotent [67, 68]. These criteria include cell/colony morphology, expression of pluripotency markers and ability to differentiate into the three primary germ layers. Once the iPS in this study were established, we analyzed their pluripotency at a cellular, molecular and functional level. At a cellular level, all the cells developed a similar colony morphology to ESC, and although not identical, they fall in the category of stable iPS colony morphology [50, 69]. At a molecular level, they expressed genes (*SOX2*, *OCT4*, *NANOG)* and proteins (OCT4, SOX2) which play a major role in inducing and maintaining the pluripotency of ESC and iPS [68]. They also displayed functional pluripotency and expressed markers associated with mesoderm (*MESP1*, *OSR1*, *HOPX*), endoderm (*GATA4*) and ectoderm (*PAX6*, *RAX*) lineages following directed differentiation. These findings demonstrate that these iPS are pluripotent and possess ESC-like characteristics. Despite the decrease in reprogramming efficiency, moving to a xeno-free protocol does not seem to have any detrimental effect on the cells after successful induction of pluripotency, as no major differences were seen between the genotype/phenotype of the iPS-PL and iPS-FBS [46]. At gene level, iPS-PL generally expressed slightly lower levels of the pluripotent markers than iPS-FBS; however, differences were insignificant. Furthermore, these differences did not seem to translate at protein level, with flow cytometry analysis revealing comparable detection of pluripotent proteins by both sets of iPS. The ability of iPS to differentiate to the three primary germ layers does not appear to be negatively affected by the use of PL, with the iPS-PL and iPS-FBS expressing comparable levels of the trilineage markers upon directed differentiation. The differentiated GF-iPS-PL expressed the markers *GATA4* and *RAX* significantly higher than the non-differentiated iPS. However, this expression was much less than the rest of the differentiated iPS, including its xenogenic counterpart. The reason for this relatively low expression is unclear. Perhaps the xeno-free protocol caused these particular cells to differentiate much slower toward endoderm and ectoderm lineages, and an increase in the duration of differentiation might result in similar expression levels to the other iPS.

With the introduction of new supplements for cell culturing protocols, it is important to ensure that no major alterations occur within the cell genome as a result of supplementation. When assessing the genetic state of human pluripotent cells, the literature shows that chromosomes 1, 12, 17, 20 and X are generally the most affected [26, 27]. Interestingly, a different set of chromosomes were more commonly affected within both iPS groups, specifically chromosomes 1, 5, 11 and 13. CNVs affecting these specific chromosomes were seen in all the iPS apart from BF-iPS-PL. Overall, both sets of iPS revealed a relatively low amount of CNVs, with the exception of the DF-iPS in both conditions, which show duplications of several segments in chromosome 1. Somatic mosaicism in the culture of fibroblasts has been shown to cause most of the genetic variation in their resultant iPS [26]. Similarly, Abyzov et al. [70] revealed in a study involving dermal fibroblasts/iPS, that 50% of the CNVs found in the iPS were present in their parental fibroblasts. This, however, is not the case with the DF-iPS in this study, as the genetic analysis revealed no CNVs in chromosome 1 of their parent fibroblasts. Hence, this particular genetic change is likely a result of the reprogramming process, or cell culture and propagation [71]. Despite their being links between aberrations in chromosome 1 to tumor formation [72], the majority of the evidence points to the harmlessness of chromosomal abnormalities in iPS. Ultimately, these genetic alterations are a common occurrence, as human pluripotent cells are often genetically unstable. Furthermore, both genomically normal and abnormal iPS can lead to teratoma formation, and there is little evidence linking the genomic abnormalities with tumorigenesis [26, 27, 63].

Analyzing the effect of cell source on iPS genotype/phenotype revealed only minor differences within each group. No particular trend was observed, however, and thus, none of the variations could be directly attributed to differences in cell source. Ultimately, these results confirm the ability to generate safe iPS from oral and dermal fibroblasts in xeno-free conditions, with quality comparable to those generated in FBS. This should allow for a smooth transition to utilizing xeno-free oral iPS for research at both the preclinical and clinical stage.

## Conclusion

For the purposes of future stem cell research and clinical translation, generating iPS in xeno-free conditions serves as a favorable strategy. When compared to FBS, the use of PL in culture media appears to lower reprogramming efficiency. Nevertheless, xeno-free dermal, buccal and gingival fibroblasts can successfully generate iPS similar to their xenogenic counterparts. The nature of fibroblast source and expansion conditions appear to have little effect on iPS genotype/phenotype. Despite having the advantage of rapid healing with minimal scar formation, oral fibroblasts proved to be more difficult to reprogram than dermal fibroblasts. Transitioning to xeno-free oral fibroblasts for generating iPS looks to be a promising approach; however, the issue of low reprogramming efficiency must be addressed in order to boost cost-effectiveness for future research and clinical use.

### Supplementary Information

**Additional file 1.** Overview of primers used for gene expression analysis

**Additional file 2.** CNV analysis data

## Data Availability

Additional data can be made available by the authors upon request. Chromosome microarray analysis data archived and shared, according to SN data policy, to NCBI’s dbVar public repository (accession: nstd231) at https://www.ncbi.nlm.nih.gov/dbvar/studies/nstd231/.

## Abbreviations

BF: Buccal fibroblasts
CNV: Copy number variation
D: Donor
DF: Dermal fibroblasts
DMEM: Dulbecco’s modified Eagle’s medium
ESC: Embryonic stem cells
FBS: Fetal bovine serum
GF: Gingival fibroblasts
GMP: Good manufacturing practice
iPS: Induced pluripotent stem cells
MSC: Mesenchymal stem cells
PL: Platelet lysate
