# Supplementary material for: Xeno-free generation of human induced pluripotent stem cells from donor-matched fibroblasts isolated from dermal and oral tissues
Source: Stem Cell Res Ther. 2023 Aug 9;14:199. doi: 10.1186/s13287-023-03403-7 (PMC10410907; doi:10.1186/s13287-023-03403-7)
Supplement: Supplementary file 1 — Additional file 1. Overview of primers used for gene expression analysis [file 13287_2023_3403_MOESM1_ESM.docx]

|  | Full name and Alias | Official symbol | Species | Assay ID |
| --- | --- | --- | --- | --- |
| Reference gene(s) | Glyceraldehyde-3-phosphate dehydrogenase; ***GAPDH*** | GAPDH | Human | Hs 99999905_m1 |
| Pluripotency gene(s) | Octamer-binding transcription factor 4; ***OCT-4; POU5F1*** | OCT-4 / POU5F1 | Human | Hs 00999632_g1 |
|  | Homeobox NANO G; ***NANO G*** | NANO G | Human | Hs 02387400_g1 |
|  | Sex determining region Y-box 2; ***SOX2*** | SOX2 | Human | Hs 01053049_s1 |
| Mesoderm genes | Mesoderm Posterior BHLH Transcription Factor 1; ***MESP1*** | MESP1 | Human | Hs 00251489_m1 |
|  | Odd-Skipped Related Transcription Factor 1; ***OSR1*** | OSR1 | Human | Hs 01586544_m1 |
|  | HOP Homeobox; ***HOPX*** | HOPX | Human | Hs 05028646_s1 |
| Endoderm genes | GATA Binding Protein 4; ***GATA4*** | GATA4 | Human | Hs 00171403_m1 |
| Ectoderm genes | Paired Box 6; ***PAX6*** | PAX6 | Human | Hs 01088114_m1 |
|  | Retinal Homeobox Protein Rx; ***RAX*** | RAX | Human | Hs 00429459_m1 |

**Table 1.** Overview of primers used for gene expression analysis.
